# Trends in HOMA-IR values among South Korean adolescents from 2007–2010 to 2019–2020: a sex-, age-, and weight status-specific analysis

**DOI:** 10.1038/s41366-023-01340-2

**Published:** 2023-07-13

**Authors:** Sujin Kim, Kyungchul Song, Myeongseob Lee, Junghwan Suh, Hyun Wook Chae, Ho-Seong Kim, Ahreum Kwon

**Affiliations:** Department of Pediatrics, Severance Children’s Hospital, Institute of Endocrinology, Yonsei University College of Medicine, Seoul, 03722 Republic of Korea

**Keywords:** Metabolic syndrome, Obesity

## Abstract

**Background/Objectives:**

An increase in obesity prevalence may lead to an increase in the HOMA-IR value. This study aimed to investigate changes in age- and sex-specific homeostasis model assessment of insulin resistance (HOMA-IR) values among South Korean adolescents, using data from the Korean National Health and Nutrition Examination Survey (KNHANES) IV, V, and VIII conducted between 2007–2010 and 2019–2020.

**Subjects/Methods:**

Overall, 4621 adolescents aged 10–18 years were evaluated, including 3473 from the 2007–2010 dataset and 1148 from the 2019–2020 dataset. The mean HOMA-IR values and percentile curves were evaluated by age, sex, and weight status.

**Results:**

The mean HOMA-IR values peaked at puberty in both sexes and further increased during puberty in the 2019–2020 dataset (boys 5.21, 95% confidence interval [CI] 4.16–6.26; girls 5.21, 95% CI 3.09–7.33) compared with the 2007–2010 dataset (boys 3.25, 95% CI 3.04–3.47; girls 3.58, 95% CI 3.31–3.85). Both groups (with normal-weight and overweight/obesity) exhibited a peak HOMA-IR value during puberty in both sexes and both datasets, although the group with overweight/obesity had a higher and wider peak age range. While the mean HOMA-IR values did not change in adolescents with normal-weight, they increased during puberty and post-puberty in boys with overweight/obesity.

**Conclusions:**

HOMA-IR values should be interpreted considering sex, weight status, and pubertal stages. In particular, during the pubertal period, insulin resistance (IR) can coexist not only due to weight-related factors but also as a result of the distinct hormonal changes characteristic of puberty. Over the 10-year period, the mean HOMA-IR values increased in the group with overweight/obesity during puberty and post-puberty, highlighting the need for active intervention to prevent metabolic complications in adolescents with overweight/obesity.

## Introduction

Insulin resistance (IR) is one of the metabolic alterations associated with obesity. IR represents relative insulin insensitivity in peripheral tissues such as the muscle, liver, and adipose tissues [1]. In the IR state, pancreatic β-cells compensate by increasing insulin secretion to maintain glucose homeostasis. Therefore, IR is closely related to abnormalities in glucose metabolism (e.g., impaired glucose tolerance and type 2 diabetes mellitus) [2] and reliably predicts the development of type 2 diabetes [3, 4]. In addition, IR is associated with the development of metabolic diseases [5]. Therefore, detecting IR in adolescents has been proposed to identify high-risk adolescents who need clinical assessment and intervention and to prevent further development of metabolic diseases.

The gold standard test for IR includes the hyperinsulinemic-euglycemic clamp [6] and minimal-model analysis frequently sampled intravenous glucose tolerance test [7]. However, because these tests are invasive, time-consuming, and expensive, they are not recommended for IR assessment in large population-based studies. The homeostasis model assessment of insulin resistance (HOMA-IR) is a relatively simple and practical method for estimating IR. The HOMA-IR has a high correlation with the hyperinsulinemic-euglycemic clamp [8–11] and is strongly correlated with IR [12]. In addition, the HOMA-IR has a positive correlation with fat mass and waist circumference [13–15] and has been proven to be an effective and simple detector of adiposity in children as a single criterion [16]. Therefore, the HOMA-IR is frequently used as a surrogate marker of IR in large-scale population-based studies [17].

The HOMA-IR value exhibits a robust correlation with the body mass index (BMI) in both adults and adolescents; typically, individuals with a higher BMI tend to exhibit higher HOMA-IR values, indicative of increased IR. However, previous studies have reported discrepancies in the reference HOMA-IR values between adults and adolescents [18]. While the HOMA-IR value does not demonstrate significant age-related variations in adults, it tends to increase during puberty, which usually occurs between the ages of 10 and 13 years [14]. It is presumed that in adolescence, variations in the secretion of sex hormones [19] and growth hormone (GH)/insulin-like growth factor I (IGF-I) [20, 21] occur as the body grows, leading to IR [22]. In other words, during adolescence, significant changes in GH/IGF-I and sex steroid levels can lead to IR, independent of obesity. Based on these findings, HOMA-IR values tend to peak during puberty and subsequently exhibit a slight decline as individuals transition into adulthood [23–25]. Moreover, girls exhibit higher HOMA-IR values than boys from pre-pubertal to pubertal ages [25–27]. Therefore, when evaluating HOMA-IR values in adolescents, the reference and cut-off value for IR should primarily be presented as a percentile, considering factors such as age, sex, and weight status [14, 15, 26].

With the increasing prevalence of obesity worldwide [28–30], it is plausible that the incidence of IR and HOMA-IR percentile values in adolescents may increase over time. However, to our knowledge, no studies have examined changes in the HOMA-IR percentile value among adolescents. Thus, this study aimed to investigate trends in HOMA-IR values among South Korean adolescents by comparing data between 2007–2010 and 2019–2020. We utilized data from the Korean National Health and Nutrition Examination Survey (KNHANES) IV, V, and VIII, which provides a nationally representative sample of 10- to 18-year-olds in South Korea. Furthermore, trends in HOMA-IR percentile values were analyzed with regard to sex, weight status, and puberty.

## Subjects and methods

### Data source

Data from the KNHANES IV, V, and VIII (2007–2010 and 2019–2020) were analyzed. Briefly, the KNHANES has been conducted periodically since 1998 by the Korea Centers for Disease Control and Prevention. It is a large, cross-sectional, and nationally representative survey of the health and nutritional status of the South Korean population.

This study was approved by the institutional review board of Yonsei University College of Medicine (approval number: 4-2022-0821) and was conducted according to the tenets of the Declaration of Helsinki.

### Anthropometry

KNHANES surveys are conducted using anthropometric measurements, including age, sex, height, weight, and BMI. Height is measured to the nearest 0.1 cm using a portable stadiometer, while weight is measured to the nearest 0.1 kg using a digital scale, with the participants wearing light clothing and no shoes. In this study, the BMI was calculated as weight in kilograms divided by the square of height in meters. Standard scores (z-scores) for the BMI were obtained for the same age and sex using the 2017 Korean Children and Adolescents Growth Chart. Underweight was defined as a BMI at the 5th percentile or below, overweight was defined as a BMI between the 85th and 95th percentiles, and obesity was defined as a BMI at the 95th percentile or above for age and sex using the 2017 Korean Children and Adolescents Growth Chart.

### Analysis dataset

This study investigated adolescents aged <19 years. Since insulin measurement was only performed for participants aged ≥10 years in the KNHANES, the study limited the participants’ age range to 10–18 years. In total, 5575 (4244 from the 2007–2010 dataset and 1331 from the 2019 to 2020 dataset) subjects aged 10–18 years were initially identified. Subjects with missing glucose or insulin levels (*n* = 553) and missing BMI values (*n* = 349) were excluded. Given that HOMA-IR may serve as a valuable surrogate measure of IR in non-diabetes adolescents [31], subjects with either a high fasting glucose level (≥110 mg/dL, diagnosed as either impaired fasting glucose or diabetes mellitus according to the World Health Organization criteria [32]) or diabetes mellitus diagnosed by doctors were excluded. A total of 52 subjects were excluded from the analysis due to a high fasting glucose level (47 subjects, 24 in the 2007–2010 dataset and 23 in the 2019–2020 dataset) and diabetes mellitus (5 subjects, 1 with type 1 diabetes and 3 with type 2 diabetes in the 2007–2010 dataset and 1 with type 1 diabetes in the 2019–2020 dataset). Finally, the analysis included 4621 subjects.

**Fig. 1:**
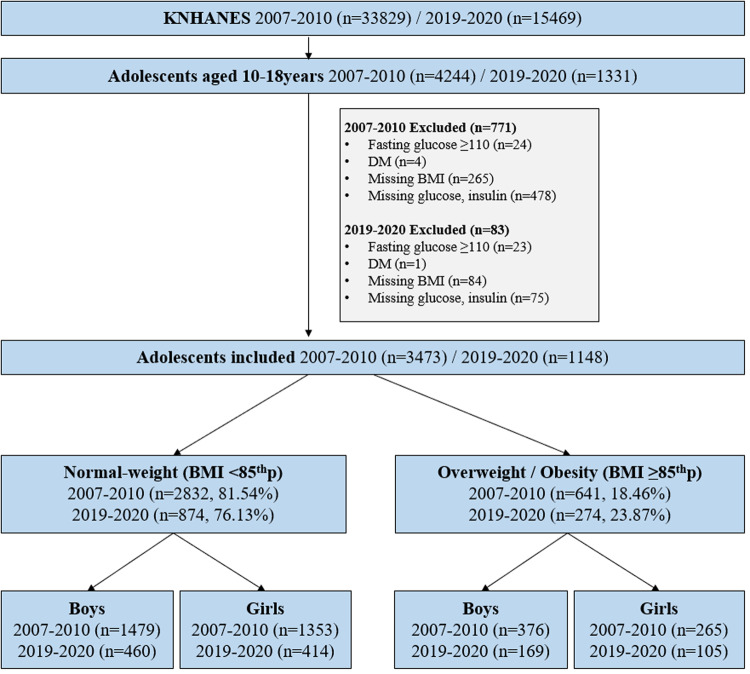
Study selection and baseline population.

As the HOMA-IR value was higher in the group with overweight/obesity than in the group with normal-weight, all subjects were divided by weight status into a group with normal-weight (i.e., BMI <85th percentile; 2832 subjects in the 2007–2010 dataset and 874 in 2019–2020 dataset) and group with overweight/obesity (i.e., BMI ≥85th percentile; 641 subjects in the 2007–2010 dataset and 274 in the 2019–2020 dataset) (Fig. 1). In addition, considering the change in the HOMA-IR value according to the pubertal stage, the subjects were also divided into three age groups as follows: pre-pubertal, pubertal, and post-pubertal [33–35]. For boys, the cut-off age was set at 12 and 15 years, considering the age of gonadarche [33] and Tanner stage 5 [34], respectively. For girls, the cut-off age was set at 13 and 16 years, considering the average age of menarche [34, 35] and Tanner stage 5 [34], respectively.

### Biochemical assays

Fasting glucose concentrations were determined according to standard procedures using an ADIVIA1650 (Siemens, Washington, DC, USA) in 2007, a Hitachi Automatic Analyzer 7600 (Hitachi, Tokyo, Japan) in 2008–2010, and Labospect 008AS (Hitachi, Tokyo, Japan) in 2019–2020. Insulin concentrations were measured using an immunoradiometric assay (INS-IRMA; Biosource, Nivelles, Belgium) with a 1470 WIZARD gamma-counter (PerkinElmer, Turku, Finland) in 2007–2010 and using an electrochemiluminescence immunoassay (ECLIA; Roche, Germany) with modular E801 (Roche, Germany) in 2019–2020. The assay detection limit was 1 μU/mL, and the intra- and inter-assay coefficients of variation were 2.2% and 6.5%, respectively. Insulin sensitivity was evaluated using the HOMA-IR index with the following equation: HOMA-IR=fasting insulin (μU/mL)×fasting glucose (mg/dL)/405.

### Statistical analyses

The means, 95% confidence intervals (CIs), and percentiles for HOMA-IR references were calculated according to age and sex in the study groups. An independent two-sample *t* test was used for continuous variables, and the Rao-Scott chi-squared test was used for categorical variables. Curves for the 3rd, 5th, 10th, 25th, 50th, 75th, 90th, 95th, and 97th percentiles were smoothed using the locally weighted scatterplot smoothing method. For all analyses, sample weights were assigned to participants to represent all adolescents in South Korea from 2007–2010 and 2019–2020. Sample weights were generated by accounting for the complex sample design that consisted of non-response rates of the target population, multistage, and posterior stratification. All analyses were performed using SAS (Version 9.4; Cary, NC, USA). All *P*-values were calculated using the two-tailed *t* test, and *P* < 0.05 was considered statistically significant.

## Results

### Baseline subject characteristics

Table 1 presents the baseline characteristics of all adolescents stratified by sex. In both sexes, the percentage of adolescents with a normal-weight had decreased, and the mean fasting glucose, insulin, and HOMA-IR values were higher in the 2019–2020 dataset than in the 2007–2010 dataset. The baseline characteristics stratified by both sex and weight status are presented in Supplementary Table 1. The mean weight, BMI, BMI z-score, fasting glucose level, insulin level, and HOMA-IR value of the group with overweight/obesity increased significantly from the 2007–2010 dataset to the 2019–2020 dataset in both sexes. Therefore, our findings suggest that the proportion of adolescents with overweight/obesity increased in 2019–2020 compared with that in 2007–2010, and the degree of obesity within the group with overweight/obesity increased, leading to a further increase in the HOMA-IR value.

**Table 1:** Baseline characteristics of total adolescents.

Sex	Boys (*N* = 2484)		*P*	Girls (*N* = 2137)		*P*
Year	2007–2010	2019–2020		2007–2010	2019–2020	
	*N* = 1855	*N* = 629		*N* = 1618	*N* = 519	
Age (years)	14.1 ± 0.1	14.3 ± 0.1	0.164	14.12 ± 0.08	14.03 ± 0.13	0.583
Height	164.8 ± 0.4	165.9 ± 0.6	0.128	157.09 ± 0.24	157.63 ± 0.45	0.290
Weight	57.7 ± 0.4	61.4 ± 0.8	**<0.001**	49.98 ± 0.35	51.32 ± 0.72	0.096
BMI (kg/m^2^)	20.96 ± 0.10	22.00 ± 0.21	**<0.001**	20.10 ± 0.11	20.46 ± 0.22	0.147
BMI (z-score)	–0.07 ± 0.04	0.25 ± 0.07	**<0.001**	–0.13 ± 0.04	–0.01 ± 0.08	0.162
BMI (percentile)						
<85p (%)	79.82	73.14	**0.001**	83.63 ± 1.12	79.82 ± 2.12	**0.007**
85p–95p (%)	10.60	10.96		9.25 ± 0.80	7.83 ± 1.29	
>95p (%)	9.59	15.89		7.12 ± 0.85	12.35 ± 1.78	
Fasting glucose (mg/dL)	89.2 ± 0.2	92.6 ± 0.4	**<0.001**	88.30 ± 0.19	91.03 ± 0.34	**<0.001**
Insulin (uIU/mL)	13.2 ± 0.2	14.8 ± 0.5	**0.004**	13.78 ± 0.22	15.22 ± 0.61	
HOMA-IR	2.94 ± 0.05	3.42 ± 0.11	**<0.001**	3.03 ± 0.05	3.49 ± 0.15	
3p	1.26	0.97		1.25	0.91	
5p	1.36	1.06		1.38	1.06	
10p	1.55	1.32		1.65	1.41	
25p	2.01	1.82		2.11	1.87	
50p	2.59	2.70		2.71	2.69	
75p	3.35	4.20		3.54	4.02	
90p	4.54	6.23		4.68	5.79	
95p	5.75	8.05		5.51	7.71	
97p	6.54	9.55		6.44	10.18	
ALT (IU/L)	18.1 ± 0.4	21.2 ± 0.8	**<0.001**	12.2 ± 0.2	13.6 ± 0.8	0.077
TG (mg/dL)	87.3 ± 1.7	90.1 ± 2.5	0.343	89.1 ± 1.6	90.4 ± 2.5	0.641
Total cholesterol (mg/dL)	154.1 ± 0.9	161.1 ± 1.4	**<0.001**	161.9 ± 0.7	166.7 ± 1.3	**0.001**
HDL (mg/dL)	48.3 ± 0.3	50.8 ± 0.5	**<0.001**	50.8 ± 0.3	53.9 ± 0.6	**<0.001**

Data are shown as the mean ± SE.

Statistically significant differences between 2007–2010 and 2019–2020 are indicated in bold font.

*ALT* alanine transaminase, *BMI* body mass index, *HDL* high-density lipoprotein, *HOMA-IR* homeostasis, *TG* tryglyceride.

### Normative age- and sex-specific HOMA-IR values and their comparison in adolescents between 2007–2010 and 2019–2020

As HOMA-IR values vary with age in adolescents, we identified the 3rd to 97th HOMA-IR percentile values in each dataset (2007**–**2010 and 2019**–**2020), stratified by sex and age (Supplementary Table 2). Table 2 presents the mean and 95% CI of the HOMA-IR values stratified by sex and age and comparison between the two datasets. The mean HOMA-IR values peaked at 12–13 years in boys and 10–13 years in girls and started to decrease thereafter in both datasets. In general, the HOMA-IR values were similar between the two datasets, except for those in boys aged 12–13 and 16 years and girls aged 12 years, which were higher in the 2019–2020 dataset than in the 2007–2010 dataset. After stratifying by pubertal stage, the mean HOMA-IR values were significantly higher during puberty in both sexes. Therefore, it is suggested that the HOMA-IR values peaked at puberty in both sexes and further increased during puberty in the 2019–2020 dataset compared with those in the 2007–2010 dataset.

**Table 2:** Comparison of the mean HOMA-IR value between the 2007–2010 and 2019–2020 datasets by age and puberty.

Sex	Age	2007–2010	2019–2020	*P*	2007–2010	2019–2020	*P*
		Mean (95% CI)	Mean (95% CI)		Mean (95% CI)	Mean (95% CI)	
Boys^§^	10	2.85 (2.64**–**3.06)	3.04 (2.57**–**3.52)	0.462	2.95 (2.78**–**3.12)	3.00 (2.66**–**3.33)	0.798
	11	3.04 (2.79**–**3.29)	2.95(2.51**–**3.38)	0.716			
	12	**3.25 (3.04–3.47)**	**4.34 (3.49–5.20)**	**0.015**	**3.18 (3.06–3.30)**	**4.16 (3.68–4.65)**	**<0.001**
	13	**3.16 (2.97–3.36)**	**5.21(4.16–6.26)**	**<0.001**			
	14	3.11 (2.93**–**3.29)	3.16 (2.72**–**3.59)	0.857			
	15	3.12 (2.80**–**3.44)	3.68 (2.97**–**4.40)	0.156	**2.78 (2.61–2.95)**	**3.12 (2.84–3.40)**	**0.041**
	16	**2.62 (2.48–2.76)**	**3.53 (2.90–4.15)**	**0.005**			
	17	2.67 (2.39**–**2.96)	2.70 (2.28**–**3.13)	0.919			
	18	2.66 (2.06**–**3.26)	2.75 (2.25**–**3.26)	0.807			
Girls^§§^	10	3.25 (2.91**–**3.59)	3.69 (2.92**–**4.46)	0.300	**3.44 (3.26–3.62)**	**4.00 (3.56–4.44)**	**0.021**
	11	3.58 (3.31**–**3.85)	3.88 (3.26**–**4.51)	0.388			
	12	**3.51 (3.27–3.76)**	**4.40 (3.65–5.16)**	**0.028**			
	13	3.27 (3.10**–**3.45)	5.21 (3.09**–**7.33)	0.073	3.06 (2.92–3.19)	3.77 (3.00–4.55)	0.072
	14	3.16 (2.93**–**3.39)	3.09 (2.58**–**3.59)	0.788			
	15	2.77 (2.54**–**3.00)	3.02 (2.52**–**3.52)	0.364			
	16	2.85 (2.67**–**3.03)	3.05 (2.46**–**3.64)	0.512	2.66 (2.49–2.82)	2.74 (2.38–3.11)	0.662
	17	2.63 (2.29**–**2.97)	2.85 (2.25**–**3.44)	0.534			
	18	2.47 (2.21**–**2.72)	2.31 (1.79**–**2.83)	0.586			

Statistically significant differences between 2007–2010 and 2019–2020 are indicated in bold font.

§: the cut-off age was set at 12 years; gonadarche and 15 years; Tanner stage 5.

§§: the cut-off age was set at 13 years; menarche and 15 years; Tanner stage 5.

*CI* confidence interval.

### Normative age- and sex-specific HOMA-IR values in adolescents with normal-weight and overweight/obesity

**Fig. 2:**
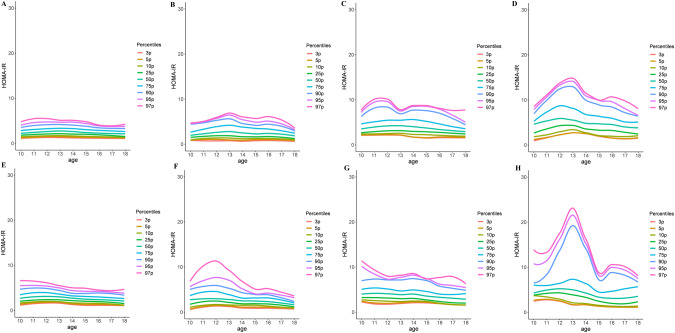
Percentiles of HOMA-IR in the groups with normal weight and overweight/obesity in the 2007–2010 and 2019–2020 datasets (LOESS). **A** Boys with normal-weight (2007–2010); (**B**) Boys with normal-weight (2019–2020); (**C**) Boys with overweight/obesity (2007–2010); (**D**) Boys with overweight/obesity (2019–2020); (**E**) Girls with normal-weight (2007–2010); (**F**) Girls with normal-weight (2019–2020); (**G**) Girls with overweight/obesity (2007–2010); and (**H**) Girls with overweight/obesity (2019–2020).

Subsequently, the 3rd to 97th HOMA-IR percentile values stratified by sex and age in either the group with normal-weight or that with overweight/obesity were identified (Supplementary Table 3-1 and 3-2). In addition, Fig. 2 shows the HOMA-IR percentiles calculated using the locally weighted scatterplot smoothing curves. Tables 3 and 4 present the means and 95% CIs of HOMA-IR values in the group with normal-weight and that with overweight/obesity, respectively. In the group with normal-weight (Table 3), the mean HOMA-IR value peaked at 12–13 years in boys and 11–12 years in girls and started to decrease thereafter. The peak mean HOMA-IR value was higher and earlier in girls than in boys in both datasets. In the group with overweight/obesity (Table 4), although the mean HOMA-IR value peaked at age 12–13 years in boys and at 10–12 years in girls in both datasets, it remained relatively high over a wide age range and decreased thereafter in both sexes and datasets. These findings suggest that both the group with normal -weight and that with overweight/obesity exhibited peak HOMA-IR values during puberty in both sexes and datasets, although the group with overweight/obesity had a relatively higher and wider peak age range than the group with normal-weight.

**Table 3:** Comparison of the mean HOMA-IR value between the 2007–2010 and 2019–2020 datasets by age in the group with normal-weight.

Sex	Age (years)	Group with normal-weight			Divided by three groups		
		2007–2010	2019–2020	*P*	2007–2010	2019–2020	*P*
		Mean (95% CI)	Mean (95% CI)		Mean (95% CI)	Mean (95% CI)	
Boys^§^	10	2.44 (2.28–2.60)	2.39 (2.07–2.72)	0.784	2.55 (2.42–2.69)	2.40 (2.18–2.61)	0.236
	11	2.64 (2.45–2.83)	2.41 (2.12–2.69)	0.170			
	12	2.83 (2.67–2.99)	2.96 (2.54–3.37)	0.573	2.82 (2.71–2.92)	3.08 (2.74–3.42)	0.151
	13	2.83 (2.68–2.99)	3.73 (2.83–4.64)	0.053			
	14	2.80 (2.61–2.98)	2.68 (2.21–3.14)	0.640			
	15	2.63 (2.42–2.84)	2.57 (2.08–3.06)	0.817	2.49 (2.31–2.66)	2.49 (2.25–2.72)	0.999
	16	**2.44 (2.29**–**2.59)**	**3.06 (2.47**–**3.64)**	**0.044**			
	17	2.33 (2.17–2.49)	2.33 (1.97–2.69)	0.998			
	18	2.53 (1.80–3.26)	2.01 (1.73–2.28)	0.189			
Girls^§§^	10	3.01 (2.68–3.33)	2.98 (2.47–3.49)	0.934	3.21 (3.05–3.38)	3.53 (3.11–3.95)	0.163
	11	3.36 (3.11–3.61)	3.65 (2.95–4.35)	0.444			
	12	3.29 (3.06–3.52)	3.90 (3.10–4.71)	0.148			
	13	3.13 (2.95–3.30)	3.66 (2.88–4.44)	0.188	2.82 (2.71–2.93)	3.00 (2.67–3.33)	0.309
	14	2.82 (2.67–2.97)	2.53 (2.21–2.85)	0.110			
	15	2.55 (2.34–2.76)	2.82 (2.32–3.31)	0.324			
	16	2.73 (2.54–2.91)	2.63 (2.35–2.91)	0.559	**2.47 (2.34**–**2.59)**	**2.18 (1.97**–**2.38)**	**0.016**
	17	2.31 (2.16–2.45)	2.02 (1.72–2.32)	0.093			
	18	**2.35 (2.07**–**2.62)**	**1.88 (1.56**–**2.20)**	**0.029**			

Statistically significant differences between 2007–2010 and 2019–2020 are indicated in bold font.

§: the cut-off age was set at 12 years; gonadarche and 15 years; Tanner stage 5.

§§: the cut-off age was set at 13 years; menarche and 15 years; Tanner stage 5.

*CI* confidence interval.

**Table 4:** Comparison of the mean HOMA-IR value between the 2007–2010 and 2019–2020 datasets by age in the group with overweight/obesity.

Sex	Age (years)	Group with overweight/obesity			Divided by three groups		
		2007–2010	2019–2020	*P*	2007–2010	2019–2020	*P*
		Mean (95% CI)	Mean (95% CI)		Mean (95% CI)	Mean (95% CI)	
Boys^§^	10	4.03 (3.60–4.47)	5.02 (3.93–6.12)	0.099	4.30 (3.85–4.76)	5.02 (4.14–5.90)	0.157
	11	4.60 (3.74–5.46)	5.00 (3.82–6.19)	0.591			
	12	**5.03 (4.37**–**5.69)**	**6.94 (5.36**–**8.52)**	**0.028**	**4.62 (4.27**–**4.98)**	**6.57 (5.63**–**7.52)**	**<0.001**
	13	**4.69 (3.97**–**5.41)**	**8.37 (6.44**–**10.29)**	**<0.001**			
	14	4.20 (3.72–4.69)	4.45 (3.40–5.50)	0.676			
	15	5.15 (4.09–6.21)	5.87 (4.65–7.10)	0.381	**4.01 (3.56**–**4.46)**	**4.95 (4.28**–**5.62)**	**0.023**
	16	**3.42 (3.05**–**3.78)**	**5.63 (3.90**–**7.35)**	**0.014**			
	17	4.02 (3.03–5.01)	3.84 (2.72–4.97)	0.814			
	18	**3.21 (2.69**–**3.74)**	**4.68 (3.59**–**5.78)**	**0.017**			
Girls^§§^	10	4.65 (3.72–5.59)	6.31 (3.81–8.81)	0.223	4.73 (4.20–5.25)	6.00 (4.83–7.18)	0.052
	11	5.42 (4.38–6.47)	5.22 (4.38–6.07)	0.769			
	12	4.37 (3.62–5.12)	6.26 (4.39–8.13)	0.065			
	13	4.07 (3.67–4.47)	11.10 (3.88–18.32)	0.056	4.29 (3.86–4.72)	7.53 (4.20–10.86)	0.058
	14	4.69 (3.83–5.55)	5.46 (3.82–7.09)	0.414			
	15	4.04 (3.36–4.72)	4.54 (2.88–6.21)	0.581			
	16	3.38 (2.90–3.87)	4.41 (2.16–6.66)	0.382	3.55 (2.91–4.20)	4.55 (3.50–5.60)	0.111
	17	4.04 (2.38–5.70)	4.76 (3.31–6.21)	0.520			
	18	3.11 (2.61–3.61)	4.35 (2.89–5.80)	0.113			

Statistically significant differences between 2007–2010 and 2019–2020 are indicated in bold font.

§: the cut-off age was set at 12 years; gonadarche and 15 years; Tanner stage 5.

§§: the cut-off age was set at 13 years; menarche and 15 years; Tanner stage 5.

*CI* confidence interval.

### Comparison of the normative distribution of age- and sex-specific HOMA-IR in adolescents with normal-weight or overweight/obesity between 2007–2010 and 2019–2020

When comparing the two datasets, it was found that the mean HOMA-IR values were similar, with the exception of those in 16-year-old boys and 18-year-old girls in the group with normal-weight. After dividing the data into three groups based on puberty, the mean HOMA-IR value was also similar between the two datasets, except in girls aged 16**–**18 years, in whom it decreased between the 2007–2010 dataset and the 2019–2020 dataset (Table 3).

In the group with overweight/obesity, the mean HOMA-IR value significantly increased from 2007–2010 to 2019–2020, with a particular increment at the ages of 12–13, 16 and 18 years in boys (Table 4). In girls, the mean HOMA-IR value increased overall for all ages in the 2019–2020 dataset compared with the 2007–2010 dataset, although this increase was not statistically significant. After dividing the data into three groups based on puberty, the mean HOMA-IR value was significantly increased during puberty and post-puberty in boys from 2007–2010 to 2019–2020. This suggests that while the mean HOMA-IR values did not change in both sexes in adolescents with normal-weight, they increased during puberty and post-puberty in boys with overweight/obesity over the 10-year period.

## Discussion

As the prevalence of obesity increases, the HOMA-IR value may change. In this study, the proportion of adolescents with overweight/obesity increased in the 2019–2020 dataset compared with the 2007–2010 dataset among South Korean adolescents. Additionally, glucose and insulin levels, as well as HOMA-IR values, were also observed to increase. When stratified by weight status, the mean HOMA-IR values were similar between the two datasets in the group with normal-weight. However, in boys with overweight/obesity, the HOMA-IR values increased from the 2007–2010 dataset to the 2019–2020 dataset, especially during puberty with significant increment. To the best of our knowledge, this is the first large-scale study to examine the difference in HOMA-IR values over a 10-year period.

The HOMA-IR value is widely recognized to be significantly influenced by the BMI [26, 27, 36–38]. Although the relationship between the HOMA-IR value and BMI is much weaker in children than in adults [39], it has been established that HOMA-IR values are naturally higher in individuals with overweight/obesity than in those with normal-weight [26], largely due to the close association between IR and obesity [26, 36–38], as well as leptin [25]. Accordingly, this study obtained percentile curves for the HOMA-IR values, not only for all subjects but also by stratifying individuals into groups with normal-weight and overweight/obesity. In this study, the means and distributions of HOMA-IR values were significantly higher in the group with overweight/obesity than in the group with normal-weight across all ages and sexes.

The HOMA-IR value of adolescents has several characteristics. First, HOMA-IR varies according to age, especially during puberty, as IR develops during this stage [26, 40–44]. In puberty, IR is associated with changes in sex steroid levels and GH/IGF-1 [19, 20, 22, 25, 26] as well as increases in adiposity [43], with insulin sensitivity decreased by approximately 25–30% [42]. Moran et al. [43] also reported that peak IR was related to the pubertal growth spurt in each sex. Second, the HOMA-IR values in girls reach their peak earlier than those in boys [14, 26]. Third, the HOMA-IR value in girls is higher than that in boys [14, 25, 27]. These observed differences in HOMA-IR values between sexes can be attributed to the earlier onset of puberty in girls, as well as a higher degree of IR exhibited in girls than in boys [43]. In accordance with previous studies [23, 24, 26, 40, 41, 43], this study demonstrated that the HOMA-IR values reach their peak during puberty and then decline toward adulthood. Furthermore, it was observed that HOMA-IR values peak at an earlier age and are higher in girls than in boys, despite slightly elevated blood glucose levels in boys as compared with girls. Notably, these characteristics of the HOMA-IR values in adolescents were found to be consistent across both the 2007–2010 and 2019–2020 datasets. These findings indicated that the normative HOMA-IR values in adolescents should consider not only sex but also their pubertal stage. To analyze the HOMA-IR values stratified by sex and pubertal status, the current study presents the HOMA-IR values for three distinct age groups in both sexes as follows: 10**–**12 years (from thelarche to menarche), 13**–**15 years (from menarche to Tanner stage 5), and 16**–**18 years (post-pubertal age) for girls [34] and 10-11 years (pre-gonadarche), 12**–**14 years (from gonadarche to Tanner stage 5), and 15**–**18 years (post-pubertal age) for boys [33, 34]. The data derived from this current study is expected to provide insights into the occurrence of IR in adolescents, as influence by pubertal hormonal characteristics and overweight/obesity. Furthermore, it is expected to contribute to the appropriate interpretation of HOMA-IR values based on specific categories.

Furthermore, it has been reported that IR associated with puberty can occur even in adolescents with overweight/obesity, irrespective of their BMI [25, 43]. Consistently, in this study, IR resulting from puberty was also observed in the group with overweight/obesity. Additionally, the age range associated with the peak HOMA-IR value was longer and fluctuated more in the group with overweight/obesity. This observation may be attributed to the coexistence of IR resulting from pubertal hormonal characteristics and an increase in adiposity. Therefore, when interpreting HOMA-IR values in adolescents with overweight/obesity, it is imperative to take into account both the contributory factors of IR. In other words, given that both contributory factors of IR can occur in adolescents with overweight/obesity, it is crucial to determine IR while considering their age and pubertal stage. In addition, considering the broader and more varied distribution of the peak HOMA-IR value in this population as compared with adolescents with normal-weight, it is important to distinguish IR resulting from increased adiposity from IR attributed to pubertal hormonal changes in this particular population. Further investigation is also required to determine the cut-off value that distinguishes between the two contributing factors of IR in adolescents with overweight/obesity. The data obtained from the group with normal-weight in this study may provide some partial assistance in this regard.

The current study revealed that over a decade, the HOMA-IR percentile value remained stable in the group with normal-weight, whereas the group with overweight/obesity showed an increase in the mean HOMA-IR value. Furthermore, an increase in the mean weight, BMI, and BMI z-score was observed in the group with overweight/obesity, and these increases appear to have contributed to an increase in both the mean and percentile values of HOMA-IR. As previously stated, the proportion of those with overweight/obesity increased during the 10-year period, which is consistent with the findings of previous studies [28, 29, 45]. Concomitant with this trend, there was a rise in both adiposity and IR within the group with overweight/obesity. These observations suggest that not only has the prevalence of overweight/obesity increased, but the risk of metabolic diseases may also have risen in the group with overweight/obesity.

The strength of the present investigation is the large sample size, wide age range, and evaluation within a decade. Given that this study is based on large-scale nationwide data, the results can be representative of South Korean adolescents, and there is no selection bias. Additionally, unlike previous studies, this study compares HOMA-IR trends over a longer period of 10 years.

However, there are some limitations. First, there is a difference in the number of subjects between the 2007–2010 and 2019–2020 datasets, and this could affect the statistical analysis. However, we conducted a weighted statistical method to overcome this issue. Second, the glucose and insulin measurement methods varied by year, with different glucose measurement devices used in 2007, 2008–2010, and 2019–2020 and different instruments for insulin level measurement used in 2007–2010 and 2019–2020. Although no studies have directly compared insulin levels between these instruments, we acknowledge that this variation in measurement instruments may have led to differences in insulin levels, which could not be adjusted for in this study. Third, since individual Tanner stages were not included in the KNHANES, the categorization into pubertal stages was based on pubertal stages of the general population rather than actual measurements. However, as the participants were representative of the general population and there was no apparent selection bias, this limitation appears to have been overcome to some extent.

In conclusion, HOMA-IR values vary by weight status, sex, and pubertal stage in South Korean adolescents. Thus, these factors should be considered in the clinical interpretation of HOMA-IR values in adolescents. Within the 10-year period, the mean and percentile values of HOMA-IR remained stable in adolescents with normal-weight, while both measures increased in adolescents with overweight/obesity. These findings suggest that there is an increasing risk of metabolic disease among adolescents with overweight/obesity over a 10-year period, highlighting the need for greater attention to be paid to this group and their specific healthcare needs.

## Supplementary information

Supplementary

## Data Availability

Correspondence and requests for materials should be addressed to Ahreum Kwon.
