# Supplementary material for: Trends in HOMA-IR values among South Korean adolescents from 2007–2010 to 2019–2020: a sex-, age-, and weight status-specific analysis
Source: Int J Obes (Lond). 2023 Jul 13;47(9):865–72. doi: 10.1038/s41366-023-01340-2 (PMC10439007; doi:10.1038/s41366-023-01340-2)
Supplement: Supplementary file 1 — Supplementary [file 41366_2023_1340_MOESM1_ESM.pdf]

Supplementary Table 1. Baseline characteristics of adolescents with normal weight and overweight/obesity

	Group with normal-weight (N=3706)						Group with overweight/obesity (N=915)					
**Sex**	**Boys (N=1939)**			**Girls (N=1767)**			**Boys (N=545)**			**Girls (N=370)**		
**Year**	**2007–2010**<br>N=1479	**2019–2020**<br>N=460	*P*	**2007–2010**<br>N=1353	**2019–2020**<br>N=414	*P*	**2007–2010**<br>N=376	**2019–2020**<br>N=169	*P*	**2007–2010**<br>N=265	**2019–2020**<br>N=105	*P*
**Age (years)**	14.2±0.1	14.4±0.1	0.266	14.1±0.1	14.0±0.1	0.546	14.0±0.2	14.3±0.2	0.336	14.3±0.2	14.2±0.3	0.860
**Height**	164.6±0.4	165.4±0.7	0.296	156.8±0.3	157.2±0.5	0.576	165.5±0.7	167.0±1.1	0.245	158.4±0.5	159.5±0.8	0.220
**Weight**	**53.7±0.4**	**55.4±0.7**	**0.041**	47.2±0.3	47.1±0.5	0.872	**73.3±0.9**	**77.9±1.5**	**0.010**	**64.1±0.8**	**67.9±1.5**	**0.021**
**BMI (kg/m^2)**	**19.57±0.08**	**19.96±0.14**	**0.013**	19.06±0.07	18.93±0.13	0.391	**26.45±0.17**	**27.56±0.32**	**0.002**	**25.42±0.22**	**26.51±0.40**	**0.017**
**BMI (z-score)**	**-0.55±0.03**	**-0.43±0.05**	**0.024**	-0.50±0.03	-0.54±0.05	0.538	**1.82±0.04**	**2.11±0.09**	**0.002**	**1.76±0.06**	**2.07±0.10**	**0.007**
**Fasting glucose (mg/dL)**	**88.9±0.2**	**92.1±0.4**	**<0.001**	**88.1±0.2**	**90.4±0.4**	**<0.001**	**90.6±0.4**	**93.7±0.6**	**<0.001**	**89.2±0.5**	**93.5±0.8**	**<0.001**
**Insulin (uIU/mL)**	11.8±0.2	11.5±0.3	0.388	12.8±0.2	12.8±0.4	0.922	**19.0±0.6**	**23.7±1.1**	**<0.001**	**18.6±0.7**	**24.8±2.3**	**0.009**
**HOMA-IR**	2.61±0.05	2.64±0.07	0.703	2.82±0.04	2.91±0.10	0.400	**4.27±0.13**	**5.53±0.26**	**<0.001**	**4.14±0.18**	**5.79±0.54**	**0.004**
3p	1.22	0.83		1.21	0.89		1.71	1.75		1.69	1.17	
5p	1.32	0.99		1.34	1.04		2.03	2.11		1.85	1.56	
10p	1.50	1.19		1.58	1.34		2.38	2.27		2.24	1.73	
25p	1.92	1.68		2.05	1.76		2.82	3.22		2.71	3.25	
50p	2.38	2.29		2.62	2.51		3.61	4.57		3.65	4.58	
75p	3.03	3.30		3.35	3.38		5.01	7.17		4.82	6.41	
90p	3.73	4.39		4.22	4.69		7.14	9.83		6.40	8.93	
95p	4.42	5.20		4.94	5.78		9.18	11.89		8.23	13.07	
97p	5.01	5.78		5.43	7.47		10.39	13.21		9.19	20.28	
**ALT (IU/L)**	**15.0±0.3**	**16.6±0.5**	**0.012**	**11.6±0.2**	**10.9±0.3**	**0.041**	30.4±1.4	33.9±2.4	0.200	**15.3±0.7**	**24.0±3.2**	**0.008**
**TG (mg/dL)**	77.9±1.4	79.6±2.2	0.512	85.7±1.6	86.2±2.6	0.865	124.5±5.5	118.9±6.1	0.494	106.5±4.7	107.1±6.4	0.936
**Total cholesterol (mg/dL)**	**150.9±0.9**	**156.9±1.4**	**<0.001**	**161.1±0.8**	**165.6±1.4**	**0.006**	166.6±1.9	172.3±2.5	0.069	165.6±2.1	170.9±2.9	0.137
**HDL (mg/dL)**	**49.4±0.3**	**52.4±0.6**	**<0.001**	**51.7±0.3**	**55.2±0.6**	**<0.001**	**44.3±0.5**	**46.3±0.6**	**0.011**	46.1±0.7	48.4±1.0	0.058

Data are shown as the mean±SE.

Statistically significant differences between 2007–2010 and 2019–2020 are indicated in bold font.

ALT = alanine transaminase, BMI = body mass index, HDL = high-density lipoprotein, HOMA-IR = homeostasis , TG = triglyceride

Supplementary Table 2. Distribution of HOMA-IR values stratified by sex and age and compared between the 2007–2010 and 2019–2020 datasets

Year	N	Age (years)	Percentiles								
			3rd	5th	10th	25th	50th	75th	90th	95th	97th
**Boys**											
2007–2010	219	10	1.21	1.31	1.57	1.98	2.53	3.42	4.65	5.43	5.97
	231	11	1.20	1.39	1.58	2.03	2.55	3.49	4.65	6.13	6.77
	248	12	1.36	1.48	1.81	2.23	2.93	3.66	5.42	5.95	6.84
	224	13	1.42	1.57	1.84	2.29	2.87	3.54	4.65	5.79	6.66
	236	14	1.25	1.47	1.63	2.22	2.81	3.70	5.07	5.85	6.45
	194	15	1.27	1.37	1.57	2.03	2.54	3.40	4.77	7.33	9.31
	181	16	1.20	1.39	1.54	2.00	2.45	3.02	3.93	4.52	5.08
	171	17	1.16	1.26	1.48	1.74	2.37	3.15	3.83	4.66	6.25
	151	18	1.15	1.20	1.36	1.71	2.29	2.80	3.55	4.02	4.59
2019–2020	83	10	0.95	0.97	1.07	1.80	2.46	4.05	5.22	6.00	7.46
	73	11	0.89	0.99	1.18	1.70	2.62	3.81	5.06	6.06	7.01
	77	12	1.23	1.44	1.77	2.19	3.34	5.37	8.30	11.34	12.38
	68	13	0.82	1.04	1.42	2.73	4.20	6.37	9.49	14.94	15.92
	74	14	0.76	1.18	1.43	1.78	2.86	3.89	5.13	6.82	7.82
	59	15	0.91	1.10	1.32	1.80	2.90	4.82	7.66	8.24	9.08
	57	16	1.07	1.08	1.37	2.00	2.89	4.46	5.49	7.59	7.94
	69	17	0.64	1.10	1.31	1.72	2.31	3.28	4.11	4.64	5.65
	69	18	0.78	0.88	1.00	1.53	2.26	3.10	5.05	6.93	7.35
**Girls**											
2007–2010	191	10	1.24	1.42	1.67	2.19	2.86	3.70	4.80	6.56	8.03
	198	11	1.45	1.57	1.89	2.41	3.32	4.35	5.30	6.51	7.68
	177	12	1.63	1.86	2.02	2.44	3.12	4.06	5.13	6.62	7.33
	202	13	1.65	1.85	2.03	2.44	3.14	3.72	4.69	5.45	5.77
	216	14	1.45	1.55	1.70	2.31	2.86	3.53	4.73	5.57	6.78
	177	15	0.98	1.23	1.58	2.05	2.54	3.08	4.01	5.72	6.11
	162	16	1.35	1.56	1.86	2.07	2.58	3.40	4.41	4.90	5.25
	161	17	1.15	1.28	1.38	1.78	2.30	2.89	3.70	4.41	4.58
	134	18	1.06	1.08	1.30	1.68	2.23	2.87	3.85	4.59	4.89
2019–2020	56	10	0.67	0.84	1.19	2.03	3.15	4.13	6.05	7.38	7.68
	69	11	1.01	1.22	1.73	2.60	3.33	4.62	5.67	7.12	9.81
	70	12	1.56	1.57	1.77	2.66	3.73	5.33	6.74	9.81	12.23
	64	13	1.34	1.44	1.71	2.20	3.25	5.09	7.90	15.44	23.86
	54	14	0.80	1.17	1.46	1.80	2.56	3.30	4.83	6.81	8.75
	51	15	0.92	1.23	1.65	1.88	2.51	3.55	4.43	7.02	8.40
	52	16	0.71	0.72	1.28	1.86	2.82	3.45	4.35	5.60	9.06
	53	17	0.74	0.78	0.98	1.60	2.06	3.02	5.85	6.71	7.23
	50	18	0.82	1.00	1.04	1.25	1.82	2.29	3.87	5.97	7.29

Supplementary Table 3-1. Distribution of HOMA-IR values in the group with normal-weight stratified by sex and age and compared between the 2007–2010 and 2019–2020 datasets

Year	N	Age (years)	Percentiles								
			3rd	5th	10th	25th	50th	75th	90th	95th	97th
Boys											
2007–2010	167	10	1.13	1.23	1.49	1.85	2.24	2.85	3.53	3.88	4.56
	181	11	1.17	1.33	1.51	1.85	2.35	3.07	3.95	5.03	6.01
	202	12	1.35	1.47	1.70	2.18	2.64	3.22	4.31	4.67	5.50
	183	13	1.30	1.53	1.75	2.09	2.75	3.37	3.91	4.44	4.71
	183	14	1.13	1.32	1.61	2.08	2.64	3.26	4.29	5.07	5.54
	159	15	1.25	1.37	1.56	1.98	2.34	3.05	3.65	4.14	4.59
	145	16	1.06	1.34	1.46	1.93	2.26	2.82	3.45	3.99	4.14
	135	17	1.11	1.23	1.42	1.68	2.21	2.84	3.37	3.66	3.73
	124	18	1.09	1.19	1.32	1.53	2.09	2.59	3.27	3.80	4.29
2019–2020	64	10	0.90	0.95	1.02	1.54	2.04	2.78	4.74	4.90	5.06
	58	11	0.64	0.98	1.14	1.70	2.23	3.07	3.98	4.19	4.24
	54	12	0.77	1.23	1.61	1.85	2.46	3.86	5.34	5.56	5.79
	47	13	0.81	0.84	1.19	1.99	3.11	4.47	6.12	7.21	7.68
	51	14	0.67	0.91	1.36	1.61	2.33	3.61	4.24	5.02	5.62
	40	15	0.65	0.99	1.16	1.56	2.21	3.42	3.91	4.49	5.29
	45	16	1.05	1.08	1.35	1.86	2.57	3.60	4.97	5.84	6.84
	50	17	0.53	0.91	1.20	1.45	2.12	2.73	3.64	4.00	5.00
	51	18	0.73	0.81	0.99	1.27	1.88	2.52	3.05	3.37	3.68
Girls											
2007–2010	160	10	1.18	1.40	1.65	2.04	2.67	3.42	4.71	5.44	6.58
	175	11	1.43	1.56	1.86	2.29	3.08	4.01	4.93	5.63	6.62
	138	12	1.70	1.86	2.00	2.36	3.07	3.97	4.94	5.27	6.03
	172	13	1.58	1.79	1.99	2.39	3.02	3.57	4.43	5.17	5.70
	177	14	1.39	1.54	1.66	2.17	2.79	3.29	3.82	4.33	4.75
	154	15	0.97	1.11	1.50	1.98	2.36	2.96	3.49	4.02	5.01
	130	16	1.25	1.51	1.74	2.03	2.52	3.19	4.26	4.63	4.81
	136	17	1.12	1.23	1.30	1.77	2.23	2.67	3.42	3.68	3.73
	111	18	1.05	1.08	1.22	1.53	2.09	2.68	3.47	3.94	4.93
2019–2020	42	10	0.64	0.72	1.06	1.73	2.74	3.73	5.14	5.92	6.83
	60	11	0.97	1.14	1.59	2.31	2.97	4.24	5.47	6.33	10.40
	54	12	1.47	1.56	1.63	2.55	2.97	4.73	5.66	7.39	10.36
	53	13	1.26	1.40	1.68	2.12	2.83	3.99	6.00	8.20	11.15
	41	14	0.80	1.03	1.39	1.80	2.50	2.77	3.48	3.99	4.56
	44	15	0.90	1.10	1.62	1.87	2.33	3.30	4.34	4.55	6.70
	43	16	0.92	1.23	1.43	1.89	2.57	3.28	3.85	4.19	4.32
	37	17	0.71	0.76	0.86	1.44	1.78	2.43	2.91	3.62	4.71
	40	18	0.74	0.93	1.04	1.22	1.70	2.13	2.47	3.26	3.55

Supplementary Table 3-2. Distribution of HOMA-IR values in the group with overweight/obesity stratified by sex and age and compared between the 2007–2010 and 2019–2020 datasets

Year	N	Age (years)	Percentiles								
			3rd	5th	10th	25th	50th	75th	90th	95th	97th
Boys											
2007–2010	52	10	2.15	2.25	2.50	2.78	3.66	4.77	5.94	6.77	7.21
	50	11	2.14	2.15	2.20	2.68	3.56	4.85	9.11	10.69	11.47
	46	12	2.02	2.12	2.62	3.29	4.61	5.54	8.06	9.24	9.77
	41	13	2.41	2.48	2.59	3.13	4.01	5.67	7.18	7.83	8.09
	53	14	1.59	1.60	2.31	2.91	4.07	5.07	6.52	6.97	7.22
	35	15	1.35	1.45	2.16	3.01	3.93	6.08	9.72	11.29	11.44
	36	16	1.92	2.05	2.32	2.71	3.11	3.82	4.82	5.17	5.43
	36	17	1.31	1.48	1.78	2.58	3.45	4.48	7.31	8.31	9.04
	27	18	1.66	1.73	2.01	2.43	2.83	3.48	4.05	4.54	7.41
2019–2020	19	10	1.08	1.42	2.23	2.89	4.83	5.79	7.70	8.74	9.46
	15	11	1.34	1.34	1.34	3.09	4.92	6.06	8.15	8.56	8.84
	23	12	2.22	2.22	3.17	4.24	5.59	8.79	12.22	13.12	13.55
	21	13	3.09	3.09	3.85	4.97	6.58	9.52	14.96	16.24	17.04
	23	14	2.16	2.16	2.20	2.91	3.53	4.57	7.96	8.98	9.56
	19	15	2.26	2.31	2.45	3.49	5.46	8.06	8.92	9.63	9.91
	12	16	1.36	1.39	1.83	3.54	4.69	5.53	10.23	11.76	11.93
	19	17	1.32	1.36	1.82	2.26	3.07	4.12	4.45	5.20	8.52
	18	18	1.59	1.69	2.13	2.66	4.19	5.54	7.38	7.54	8.49
Girls											
2007–2010	31	10	2.32	2.43	2.70	3.09	3.78	4.76	6.84	10.24	11.38
	23	11	1.80	2.10	2.71	3.72	4.86	6.21	7.53	8.07	9.18
	39	12	1.48	1.74	2.31	2.61	3.60	4.86	8.14	8.67	9.24
	30	13	2.36	2.36	2.44	3.41	4.08	4.50	5.46	5.71	6.14
	39	14	1.70	2.06	2.42	2.82	4.02	5.27	9.01	10.16	10.63
	23	15	2.33	2.34	2.46	2.82	3.60	4.51	6.40	6.79	6.91
	32	16	1.87	1.92	2.04	2.42	3.13	3.88	5.43	5.45	5.72
	25	17	1.39	1.40	1.49	1.88	3.35	4.06	6.17	7.60	11.94
	23	18	1.68	1.69	1.71	2.10	2.78	4.10	4.56	4.88	4.88
2019–2020	14	10	2.20	2.42	3.65	3.88	4.31	6.20	7.81	12.65	16.12
	9	11	3.57	3.57	3.57	4.01	4.90	5.64	5.91	7.29	7.92
	16	12	2.11	2.24	2.84	4.30	5.07	6.03	8.74	12.62	15.11
	11	13	1.71	1.76	2.23	4.19	5.32	7.88	27.63	29.20	29.83
	13	14	1.60	1.60	1.60	2.77	4.02	6.51	8.94	10.40	12.12
	7	15	1.66	1.66	2.16	3.14	3.55	4.11	7.29	8.13	8.47
	9	16	0.72	0.72	0.72	1.36	2.89	4.43	10.43	11.48	11.70
	16	17	1.61	1.61	1.75	2.22	3.36	6.31	7.23	8.81	10.55
	10	18	0.99	1.03	1.27	2.28	3.50	5.29	7.00	7.61	7.90

Supplementary Table 4. Distribution of HOMA-IR values stratified by sex and age and compared between the 2007–2010 and 2019–2020 datasets by age group

Year	N	Age (years)	Percentiles								
			3rd	5th	10th	25th	50th	75th	90th	95th	97th
Boys											
2007–2010	450	10–11	1.22	1.34	1.60	2.00	2.54	3.46	4.65	5.82	6.63
	708	12–14	1.34	1.52	1.74	2.23	2.88	3.69	5.08	5.94	6.52
	697	15–18	1.22	1.32	1.47	1.92	2.40	3.08	4.00	5.06	6.28
2019–2020	156	10–11	0.95	0.99	1.18	1.70	2.51	3.90	5.29	6.06	7.84
	219	12–14	0.83	1.24	1.50	2.13	3.26	5.02	7.82	10.11	12.94
	254	15–18	0.97	1.05	1.26	1.77	2.47	3.82	5.52	7.50	8.06
Girls											
2007–2010	566	10–12	1.43	1.57	1.86	2.32	3.06	4.07	5.18	6.61	7.74
	595	13–15	1.29	1.53	1.78	2.24	2.81	3.51	4.54	5.61	6.15
	457	16–18	1.10	1.25	1.40	1.90	2.40	3.05	4.08	4.66	5.20
2019–2020	195	10–12	0.99	1.27	1.58	2.55	3.39	4.90	6.07	7.69	10.71
	169	13–15	1.12	1.38	1.59	2.00	2.62	3.81	6.08	8.72	12.26
	155	16–18	0.73	0.88	1.10	1.61	2.13	3.10	5.01	7.12	7.39

Supplementary Table 5-1. Distribution of HOMA-IR values in the group with normal-weight stratified by sex and age and compared between the 2007–2010 and 2019–2020 datasets by age group

**Year**	**N**	**Age (years)**	**Percentiles**								
			**3rd**	**5th**	**10th**	**25th**	**50th**	**75th**	**90th**	**95th**	**97th**
Boys											
2007–2010	348	10–11	1.16	1.29	1.51	1.85	2.33	2.96	3.78	4.51	5.43
	568	12–14	1.30	1.47	1.69	2.11	2.65	3.29	4.11	4.68	5.43
	563	15–18	1.19	1.26	1.43	1.81	2.25	2.84	3.46	3.96	4.48
2019–2020	122	10–11	0.93	0.96	1.05	1.60	2.19	3.00	4.12	4.83	4.89
	152	12–14	0.80	1.02	1.36	1.78	2.69	4.06	5.23	6.25	6.92
	186	15–18	0.82	0.99	1.13	1.56	2.20	2.95	4.08	4.97	5.45
Girls											
2007–2010	473	10–12	1.40	1.56	1.78	2.22	2.91	3.84	4.84	5.53	6.60
	503	13–15	1.25	1.42	1.69	2.15	2.69	3.28	3.88	4.71	5.39
	377	16–18	1.09	1.19	1.38	1.85	2.32	2.88	3.67	4.38	4.65
2019–2020	156	10–12	0.97	1.14	1.56	2.20	2.95	4.18	5.50	6.89	7.83
	138	13–15	1.03	1.27	1.49	1.88	2.51	3.27	4.57	6.01	8.08
	120	16–18	0.75	0.87	1.07	1.47	1.94	2.61	3.45	3.88	4.34

Supplementary Table 5-2. Distribution of HOMA-IR values in the group with overweight/obesity stratified by sex and age and compared between the 2007–2010 and 2019–2020 dataset by age group

Year	N	Age (years)	Percentiles								
			3rd	5th	10th	25th	50th	75th	90th	95th	97th
Boys											
2007–2010	102	10–11	2.15	2.18	2.27	2.76	3.62	4.77	6.57	8.99	10.68
	140	12–14	1.71	2.17	2.57	3.15	4.15	5.47	7.09	8.33	9.08
	134	15–18	1.57	1.74	2.12	2.67	3.18	4.38	7.28	9.38	10.67
2019–2020	34	10–11	1.15	1.27	2.24	3.12	5.06	6.02	8.04	8.95	9.62
	67	12–14	2.20	2.24	2.90	3.81	5.41	8.14	12.17	14.10	15.07
	68	15–18	1.57	1.82	2.19	2.91	4.18	5.62	8.07	10.18	11.41
Girls											
2007–2010	93	10–12	2.27	2.31	2.53	3.00	3.95	5.34	8.19	9.16	10.85
	92	13–15	2.31	2.35	2.48	2.91	3.91	4.90	6.51	8.01	9.09
	80	16–18	1.46	1.53	1.80	2.27	3.15	4.11	5.43	6.11	6.57
2019–2020	39	10–12	2.21	2.54	3.55	4.20	5.04	5.90	8.11	11.40	17.92
	31	13–15	1.60	1.66	2.17	3.55	4.66	7.55	15.08	25.73	28.46
	35	16–18	0.72	0.81	1.41	1.93	3.56	6.32	7.39	11.33	12.02
